# “Non-healing” claw horn lesions in dairy cows: Clinical, histopathological and molecular biological characterization of four cases

**DOI:** 10.3389/fvets.2022.1041215

**Published:** 2022-10-19

**Authors:** Maher Alsaaod, Jim Weber, Tim Jensen, Sabine Brandt, Corinne Gurtner, David Devaux, Eveline Studer, Adrian Steiner

**Affiliations:** ^1^Clinic for Ruminants, Vetsuisse Faculty, University of Bern, Bern, Switzerland; ^2^Center for Diagnostic, Technical University of Denmark, Kongens Lyngby, Denmark; ^3^Section for Pathobiological Sciences, University of Copenhagen, Copenhagen, Denmark; ^4^Research Group Oncology, Equine Clinic of Surgery, Department of Companion Animals and Horses, University of Veterinary Medicine, Vienna, Austria; ^5^Department of Infectious Diseases and Pathobiology, Vetsuisse Faculty, Institute of Animal Pathology, University of Bern, Bern, Switzerland; ^6^Department of Farm Animals, Vetsuisse Faculty, University of Zurich, Zürich, Switzerland

**Keywords:** bovine digital dermatitis, dairy cow, fluorescent *in situ* hybridization, treponemes, PCR

## Abstract

The increasing prevalence of bovine digital dermatitis (BDD) contributes to a higher occurrence of secondary infections of exposed corium with *Treponema* spp. in bovine claws. “Non-healing” claw horn lesions (NHL) clinically resemble BDD lesions. They are severe, cause chronic lameness, and may persist for several months. They poorly respond to standard treatments of BDD and represent a serious welfare issue. In this study, four cases of NHL were classified clinically either as BDD-associated axial horn fissures (BDD-HFA; *n* = 3) or BDD-associated sole ulcer (BDD-SU; *n* = 1). In all four cases, pronounced multifocal keratinolysis of the stratum corneum, ulceration, and severe chronic lymphoplasmacytic perivascular to interstitial dermatitis were observed. All lesional samples tested positive for *Treponema* spp., *Fusobacterium* (*F*.) *necrophorum*, and *Porphyromonas* (*P*.) *levii* by PCRs. BDD-HFA lesions contained *Treponema pedis* as revealed by genetic identities of 93, 99, and 100%. Treponemes in the BDD-SU lesion were 94% homologous to *Treponema* phylotype PT3. Fluorescent *in situ* hybridization (FISH) revealed extensive epidermal infiltration by treponemes that made up > 90% of the total bacterial population in all four lesions. FISH also tested positive for *P. levii* and negative for *F. necrophorum* in all four cases, whilst only one BDD-HFA contained *Dichelobacter nodosus*. Our data point to BDD-associated treponemes and *P. levii* constituting potential etiological agents in the development of “non-healing” claw horn lesions in cattle.

## Introduction

Bovine digital dermatitis (BDD) was first described by Cheli and Mortellaro in Italy in 1974 (1). Since then, the prevalence of the disease has continuously increased in many countries (2, 3). The etiology of BDD is polymicrobial (4), with specific *Treponema* spp. being most commonly associated with disease development and progression (5–7). BDD typically involves the digital skin and is often classified using the M-stage scoring system (8). An increase in the prevalence of BDD also contributes to a higher occurrence of secondary infections of the exposed corium by *Treponema* spp. (9, 10). Clinically, “non-healing” claw horn lesions (NHL) are similar to BDD lesions and characterized by a moist, topical granular appearance and pungent fetid smell (11, 12). In contrast to the classical forms of non-infectious claw horn diseases, Evans et al. (11) reported that NHL are typically more severe, cause chronic lameness, and poorly respond to state-of-art BDD treatment strategies. NHL may persist for many months, and thus, can seriously compromise the welfare of affected animals (13). NHL is also known as “BDD-associated claw horn lesion”. Today, a curative treatment strategy for NHL is available (13–15). The latter consists of extensive debridement of loose horn and infected corium under local anesthesia, followed by topical application of copper sulfate, antibiotics (e.g., oxy-/ chlortetracycline), or salicylic acid under a bandage, and application of a block on the sound partner claw (14).

Molecular studies provide evidence of a strong association of three distinct phylogroups of BDD-associated *Treponema* spp. with NHL (11, 16, 17). The objective of the herein-reported study was to characterize four NHL cases in Swiss dairy cows with respect to their clinical appearance, histopathological features, and bacterial infection by a combination of histopathological and molecular biological techniques.

## Materials and methods

### Case history

Two NHL-affected cows from the same farm were presented at the Clinic of Ruminants, Vetsuisse Faculty, University Zurich, one NHL case was diagnosed at the Clinic of Ruminants, Vetsuisse Faculty, University of Bern, and one NHL case was detected during a herd health visit at a dairy farm located in the canton of Fribourg, Switzerland.

Respiratory rate (30, 32, and 32) was assessed by observation of thoracic movements, heart rate (92, 72, and 60) was assessed by cardiac auscultation in cows 1, 2, and 3, respectively. The rectal body temperature (38.4, 38.2, 38.4, and 38.8°C) was measured with a rectal thermometer in cows 1, 2, 3, and 4, respectively.

All cows showed mild to moderate lameness for several weeks (locomotion score ranged between 2 and 3; Table 1) according to Sprecher et al. (19), with 1 = non-lame to 5 = severely lame. After applying a wooden block on the partner claw and cleansing of the affected area with 1%-povidone-iodine or Octenidine solution (Octenisan^®^ Schülke und Mayr AG, Frauenfeld, Switzerland), all lesions were treated by extensive surgical debridement including the removal of loose horn and removal of granulation tissue under three-point local anesthesia using lidocaine hydrochloride (Lidocaine 2%, Streuli Tiergesundheit AG, Uznach, Switzerland). Thereafter, a wound dressing with diluted oxytetracycline (Engemycin^®^ 10% injectable solution, MSD Animal Health GmbH, Luzern, Switzerland) or an octenidine ointment (Octenisept^®^ Gel, Schülke und Mayr Ag, Frauenfeld, Switzerland) was administered topically onto all BDD-HFA, and bandages were applied. Treatment was repeated every 2–3 days until the wound healed, and then at weekly intervals until defects were covered by a sufficient and stable new horn.

**Table 1:** Locomotion score and results of bacteriological and histopathological investigations as well as fluorescent *in situ* hybridization of biopsies collected from four non-healing lesions associated bovine digital dermatitis (BDD).

	**Cow 1**	**Cow 2**	**Cow 3**	**Cow 4**
**Lesion type**	**BDD-HFA**	**BDD-HFA**	**BDD-HFA**	**BDD-SU**
Locomotion score	2	2	3	3
**Bacteriological evaluation**				
TT PCR^a^ Amplified sequence {[GenBank accession no.] best match (identity in %)}	+ [KJ206528] *T. pedis* (99)	+ [KR025849] *T. pedis* (100)	+ [KJ206531] *T. pedis* (93)	+ [AM942447] PT3 (94)
*D. nodosus* PCR	+	-	-	-
*F. necrophorum* PCR	+	+	+	+
*P. levii* PCR	+	+	+	+
**Histopathological evaluation**				
Keratinolysis score/dermatitis score (0–3)^b^	3/3	3/3	3/3	3/3
Spirochete load score (0–3)^c^	3	1	1	1
**Fluorescent** ***in situ*** **hybridization**				
*Treponema* spp. score^d^	3	3	3	3
*Treponema* (*T*.) phylotypes (score; 0–3)	*T. phagedenis* (3), *T. pedis* (3), *T. medium* (2)	*T. pedis* (3)	n.a.	n.i.
*D. nodosus* score (0–3)	2	0	n.a.	0
*F. necrophorum* score (0–3)	0	0	n.a.	0
*P. levii* score (0–3)	3	3	n.a.	3

BDD-associated axial horn fissure (BDD-HFA; n = 3) and BDD-associated sole ulcer (BDD-SU; n = 1).

^a^Allows for detection of *Treponema* spp. and *Tannerella* spp. by using universal Treponema primers 5'/3 TT (PCR +: presence and –: absence).

^b^Keratinolysis was graded as follows: 0, none; 1, focal; 2, multifocal; and 3, extensive; chronic dermatitis was graded as follows: 0, no changes present; 1, mild chronic; perivascular; lymphoplasmacytic dermatitis; 2, moderate chronic; perivascular, lymphoplasmacytic dermatitis; 3, severe chronic, perivascular, lymphoplasmacytic dermatitis.

^c^Number of spirochetes visualized by Warthin-Starry stain was graded as follows: 0, none visible; 1, minimal amount; 2, moderate amount; 3, high amounts.

^d^Hybridization signal was graded according to Klitgaard et al. (18): 0, no hybridization; 1, sparse hybridization; 2, moderate hybridization; and 3, strong hybridization.

*D., Dichelobacter; F., Fusobacterium;* n.a., not available; n.i., not identified; *P., Porphyromonas*.

Management of the BDD-SU consisted of removal of granulation tissue under regional intravenous anesthesia using 20 mL of lidocaine hydrochloride, application of povidone-iodine ointment (Betadine^®^, Covetrus AG, Lyssach, Switzerland) under the bandage and weekly bandage changes until defects were covered by new horn.

### Sample collection, processing, and DNA extraction

Affected feet were washed with 1%-povidone-iodine or octenidine solution. Subsequently, lesional tissue was obtained in the course of therapeutic debridement. Each tissue sample was longitudinally bidissected on a sterile petri dish using sterile forceps and #11 scalpel blades.

Sample processing for both molecular biological and histopathological analyses was done as described previously by Alsaaod et al. (20). For histopathological evaluation, one sample section was fixed in 10% neutral buffered formalin, embedded in paraffin, cut, and stained with hematoxylin and eosin or with the Warthin-Starry stain.

From the other sample section, DNA was extracted using a DNeasy Blood and Tissue kit (Qiagen, Hilden, Germany) according to the manufacturer's protocols. Purified DNA samples were stored at −20°C until further use.

### Histopathological evaluation

Histopathological evaluation was performed according to Read and Walker (21) modified by Klitgaard (18) to classify epidermal and dermal changes. Characteristic BDD-associated changes commonly include (i) a focally circumscribed hyperplastic epidermis with or without parakeratotic papillomatous proliferation, (ii) loss of the stratum granulosum, and/or iii) dermal inflammation. Histopathological scoring also took degrees of keratinolysis and chronic, perivascular, lymphoplasmacytic dermatitis into account. Keratinolysis was graded as follows: 0 = none, 1 = focal, 2 = multifocal, and 3 = extensive. Chronic dermatitis was classified as follows: 0 = no changes present, 1 = mild chronic, perivascular, lymphoplasmacytic dermatitis, 2 = moderate chronic, perivascular, lymphoplasmacytic dermatitis, and 3 = severe chronic, perivascular, lymphoplasmacytic dermatitis. The number of spirochetes visualized by Warthin-Starry stain was semi-quantitatively categorized as 0 = non-visible, 1 = minimal amount, 2 = moderate amount, and 3 = high amount of spirochetes.

### PCR assays and sequencing

DNA extracts were screened for the presence of treponemal DNA using consensus “total” *Treponema* (TT) primers 5′/3′ (TT-PCR) designed by Moe et al. (22) according to an optimized protocol (23), which is described in detail by Alsaaod et al. (20). For each PCR, sterile water, confirmed *Treponema*-free equine skin DNA, and *Treponema* DNA-positive BDD DNA were co-analyzed as no-template, negative, and positive control, respectively. Amplification products (corresponding to the most abundant spirochetal DNA per sample) were analyzed by gel electrophoresis and visualized by ethidium bromide staining, with a GeneRuler 100 bp DNA ladder (ThermoScientific, Vienna, Austria) serving as molecular weight marker. Amplicon aliquots of anticipated size were gel-purified using a QIAex II gel extraction kit (Qiagen, Hilden, Germany) according to the manufacturer's instructions and then subjected to direct bidirectional sequencing (Eurofins Genomics) using *Treponema* primers 5′/3′ TT (10 pmol/μl). After alignment of positive and negative strand sequences, only the 5'/3'matching DNA sequence was subjected to BLAST alignment to search for highly homologous bacterial sequences (https://blast.ncbi.nlm.nih.gov/Blast.cgi).

Furthermore, NHL-derived DNA aliquots were subjected to specific *Dichelobacter (D.) nodosus*, and *Fusobacterium (F.) necrophorum* PCRs as described by Sullivan et al. (24). For detection of *Porphyromonas (P.) levii*, primers for amplification of a 16s rRNA gene fragment were designed according to *P. levii* reference strain GU454798. The 5' primer sequence (F-Primer 677) was 5'-AAGGCAGCTTACAAAAGTGTA-3' and the 3' primer sequence (R-Primer 812) was 5'-TTTCGCTTGAGAGCATACAT-3'. Each PCR contained 10 μl GoTaq^®^ Green Master Mix (Promega AG, Switzerland), 0.1 μl of each primer, and 1 μl of PCR template. The reaction mixtures were heated to 95 °C for 5 min, then cycled 35 times at 95 °C for 1 min, 54 °C for 1 min, and 72 °C for 2 min, followed by incubation at 72 °C for 5 min. As a positive control, skin samples from sheep (*D. nodosus*) and cattle (*F. necrophorum* and *P. levii*) were included, and sterile water was used as no-template control.

### Fluorescent *in situ* hybridization

For FISH analysis, serial 4-μm sections were prepared from formalin-fixed, paraffin-embedded biopsies and hybridized as described previously (25). In brief, hybridization was carried out at 45 °C for 16 h and a final probe concentration of 5 ng/μL. After hybridization, the slides were washed three times in pre-warmed (45 °C) hybridization buffer for 3 minutes and subsequently with washing buffer at the same time intervals. The sections were rinsed in water, air dried, and mounted in Vectashield (Vector Laboratories Inc., Burlingame, CA, USA) for fluorescence microscopy. The probe for domain bacteria was 5' labeled with fluorescein isothiocyanate (FITC) and all other bacteria probes were 5' labeled with the isothiocyanate derivative Cy3 (Eurofins MWG Operon, Ebersberg, Germany).

The oligonucleotide probes used in this study are listed in Supplementary Table S1 and included probes specific for the domain *Bacteria, F. necrophorum, D. nodosus, P. levii*, the genus *Treponema*, and four different *Treponema* phylotypes (i.e., *T. pedis, T. phagedenis, T. medium*, and *T. refringens*). The hybridization signal was scored from 0 to 3 according to Klitgaard et al. (18), where 0 = no hybridization, 1 = sparse hybridization, 2 = moderate hybridization, and 3 = strong hybridization.

## Results

### Clinical findings

**Figure 1:**
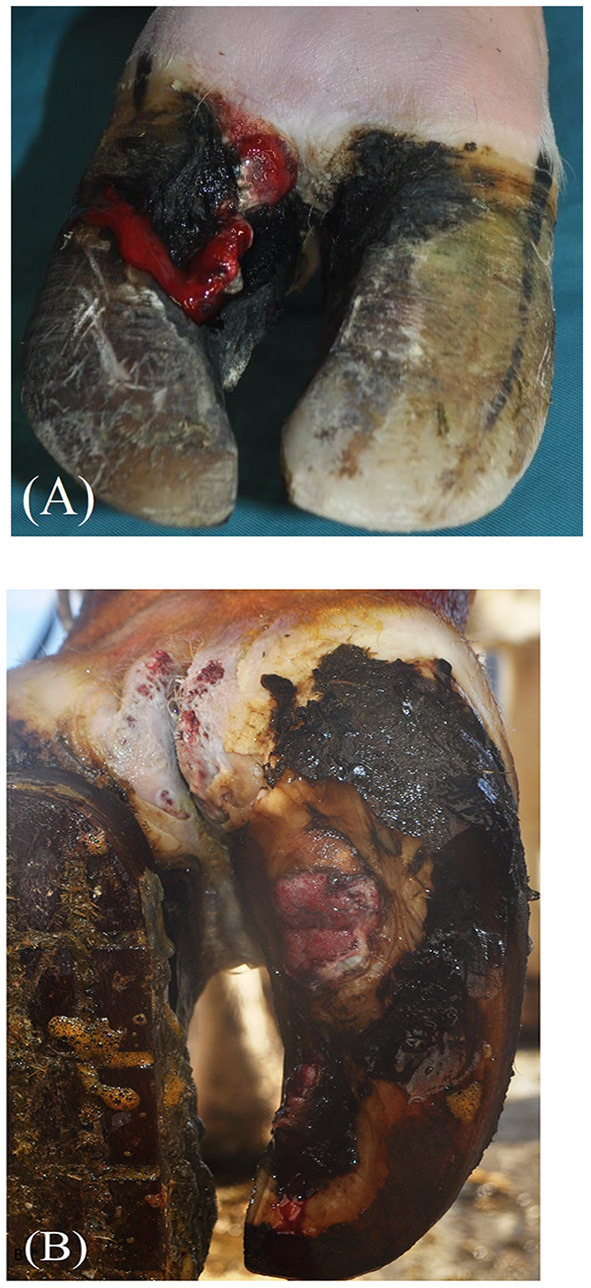
Gross pathology of bovine digital dermatitis (BDD)-associated axial horn fissure [BDD-HFA; **(A)**] and BDD-associated sole ulcer (BDD-SU) before treatment **(B)**.

BDD-HFA and BDD-SU lesions were severe, penetrating the horn capsule, and involving the corium. Lesions exhibited hypergranulation tissue covering the axial horn fissure (*n* = 3) or the plantar sole area (*n* = 1) and were classified according to the International Committee for Animal Recording Claw Health Atlas Kofler et al., (13) as BDD-associated axial horn fissures (BDD-HFA; Figure 1A), and BDD-associated sole ulcer (BDD-SU; Figure 1B), respectively.

Figure 1 exemplarily depicts the gross pathological features of one case of BDD-HFA (A) and BDD-SU (B). The treatment duration ranged between 2 and 4 weeks until defects were covered by a sufficient and stable new horn.

### Histopathological findings

**Figure 2:**
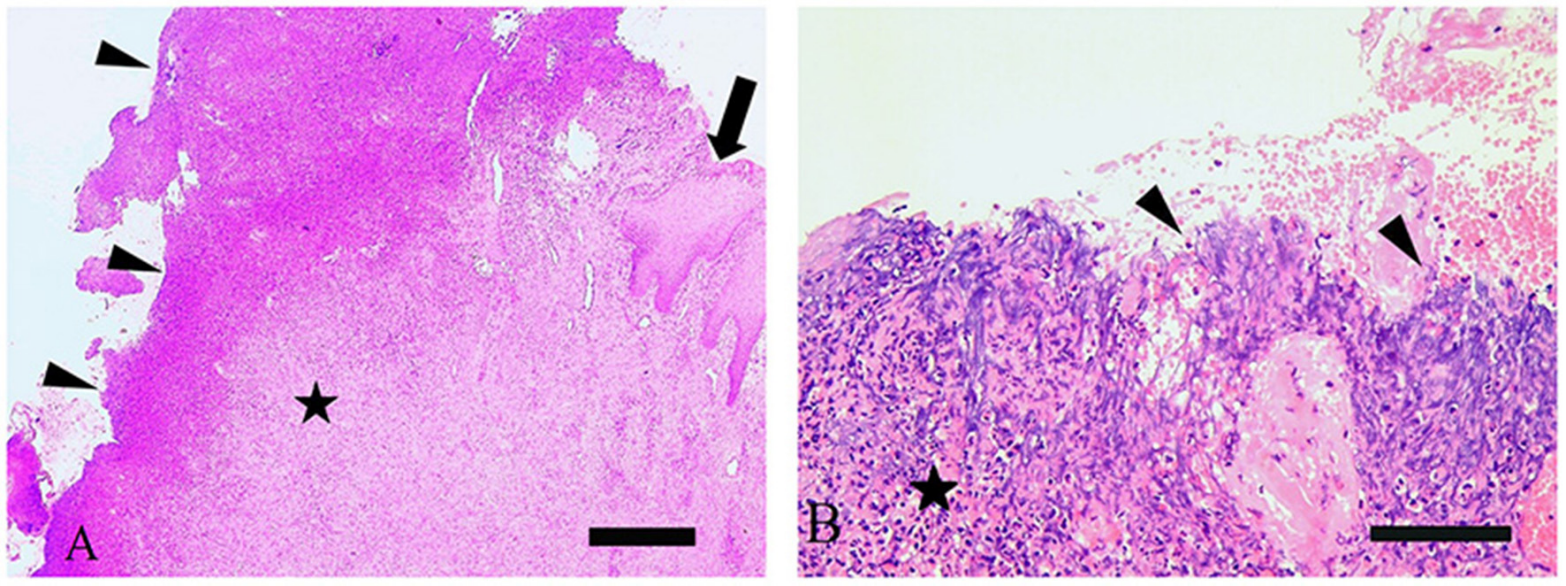
**(A)** Histological investigation of bovine digital dermatitis (BDD)-associated axial horn fissure revealed severe fibrosis (star) of dermis, focal areas with nests of epidermis (arrow) and large areas with ulceration covered by cellular debris (arrowheads). H&E, bar 500 μm. **(B)** BDD-associated sole ulcer covered by cellular debris (arrowheads), superficial bleeding and severe fibrosis (stars) of dermis. H&E, bar 200 μm.

All four lesions exhibited severe multifocal keratinolysis of the stratum corneum with ulceration (Figures 2A,B) as well as loss of the stratum granulosum. In three cases, the epidermis was acanthotic. Warthin-Starry stain pointed to the presence of spirochetes on the surface and within the epidermis in all samples, with spiral bacteria being observed (Table 1). All lesions also displayed severe, chronic lymphoplasmacytic perivascular to interstitial dermatitis.

### PCR detection and sequencing results

All lesions tested positive by total *Treponema* PCR. Amplicon sequencing and subsequent BLAST alignment resulted in the identification of three *T. pedis* sequences from BDD-HFA lesions, showing 99% identity with *T. pedis* strain G2JD, 100% identity with *T. pedis* strain DD3F, and 93% identity with *T. pedis* strain G9JD, respectively. The amplicon detected from the BDD-SU lesion was 94% homologous to *Treponema* phylotype PT3 AM942447 (18). All lesions also tested positive for *F. necrophorum* and *P. levii*, whilst only one BDD-HFA scored positive for *D. nodosus* (Table 1). TT PCR from no-template, negative, and positive control yielded the expected results, thus confirming the authenticity of obtained results.

### FISH results

**Figure 3:**
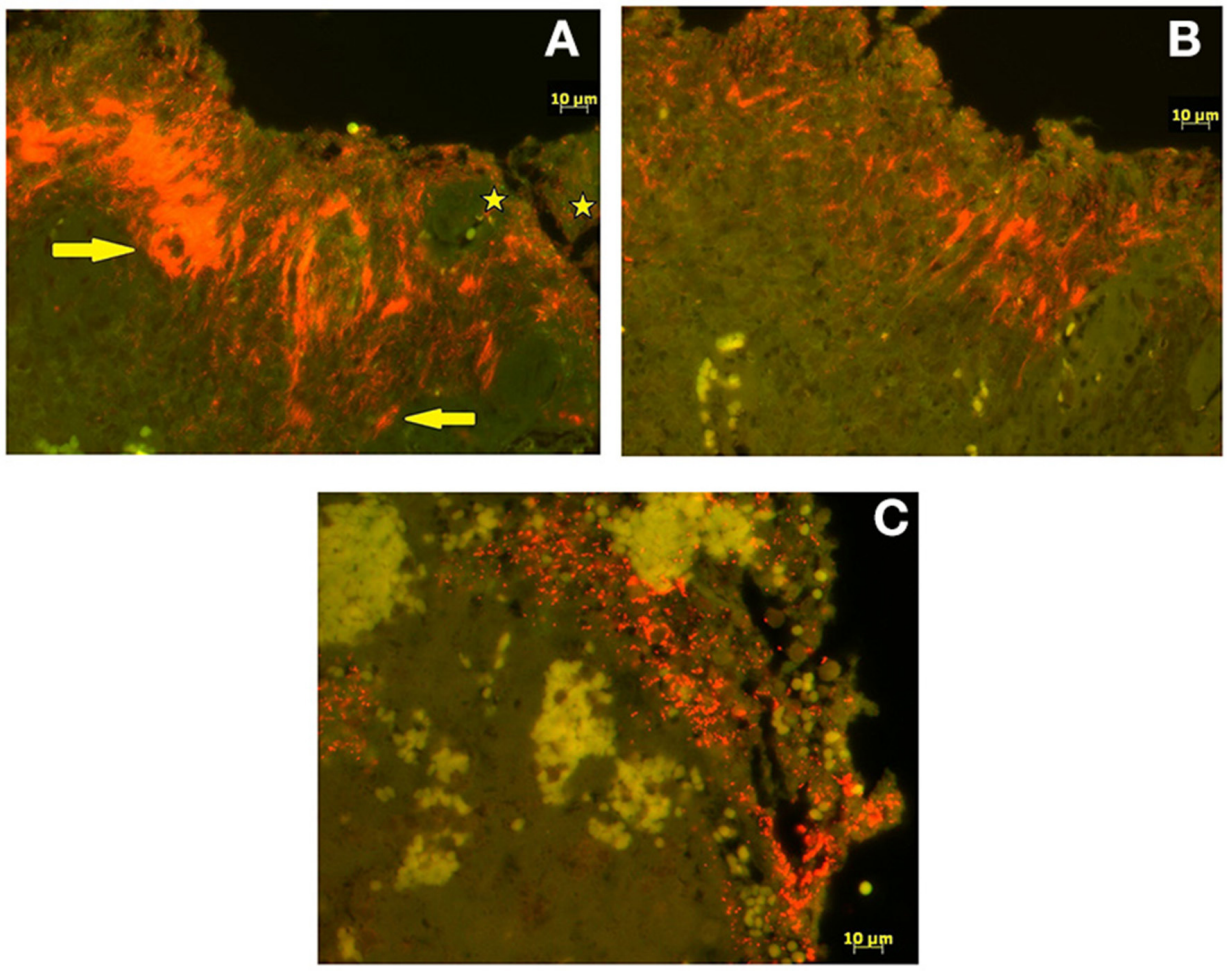
Fluorescent *in situ* hybridization. **(A)** Double fluorescent *in situ* hybridization with probe for genus *Treponema* (Cy3 labeled; orange) and for domain Bacterium (fluorescein labeled; bright green). *Treponema* organisms (arrows) infiltrating deep into the epidermis whereas other bacteria (stars) are seen superficially. **(B)** Single fluorescent *in situ* hybridization (Cy3 labeled; orange) for species specific oligonucleotide probes for *Treponema pedis*. **(C)** Single fluorescent *in situ* hybridization (Cy3 labeled; orange) for species specific oligonucleotide probes for *Porphyromonas levii*.

FISH analysis revealed severe, extensive epidermal infiltration (score 3) by treponemes that made up 90% of the total bacterial population in all four cases (Figure 3A). Additional FISH analysis of *Treponema* spp. positive biopsy samples revealed a mixed infection with *T. phagedenis, T. pedis*, and *T. medium* in one BDD-HFA, whereas another BDD-HFA was only infected by *T. pedis* (Figure 3B). BDD-SU tissue tested FISH positive for the genus *Treponema* but was negative for all four *Treponema* phylotypes assessed. Moderate hybridization for *D. nodosus* was only noted in one case of BDD-HFA. All three BDD-HFA lesions exhibited a strong hybridization signal for *P. levii* (Figure 3C). *F. necrophorum* could not be detected in any of the lesions (Table 1).

## Discussion

Reports on the prevalence and biopathological features of bovine NHL have increased over the past years. Yet, many aspects of the etiopathogenesis of the disease remain unclear. To our knowledge, to date, NHL has not been histopathologically analyzed in combination with molecular biological techniques.

All four BDD-NHL-affected dairy cows originated from herds with a history of BDD and were kept in a freestall systems. Endemically BDD-affected herds are described to be at higher risk of developing NHL (9, 10, 26).

Severe multifocal keratinolysis of the stratum corneum, ulceration and severe, chronic lymphoplasmacytic perivascular to interstitial dermatitis in concert with spirochetal colonization were the predominant histological features noted in all four cases. These findings are consistent with the typical pathological features of BDD (18, 27).

*Treponema* spp. have been reported as a cause of contagious ovine digital dermatitis (CODD) in sheep (28, 29). In small ruminants and wildlife elk, treponemal infections typically lead to dermatitis along the sole, which can result in complete hoof avulsion in severe cases (29, 30). More recently, BDD-associated *Treponema* spp. were detected in captive European bison (*Bison bonasus*) in Switzerland (31). In agreement with Evans et al. (11) and Sykora et al. (17), treponemal DNA was detected in all cases of NHL in our study. In the study by Sykora et al. (17), *T. medium* was almost exclusively found in BDD-WLD and BDD-SU, whereas *T. pedis* was equally detected in common BDD lesions and NHL. As a result, it was proposed that *T. medium* might play a key role in the pathogenesis of BDD-WLD and BDD-SU. In the present study, *T. medium* was only identified in one case of BDD-HFA. Two of the four cases were positive for *T. pedis* as confirmed by FISH and PCR followed by amplicon sequencing. Therefore, further research is needed to clarify the role of different *Treponema* spp. by screening different types of NHL vs. common BDD lesions using *Treponema* spp.-specific detection methods.

*P. levii* is a pleomorphic, gram-negative, anaerobic, rod-shaped bacterium possibly associated with bovine metritis (32), bovine necrotic vulvovaginitis (33), BDD (34) and bovine interdigital phlegmon (35). Whilst there is a lack of information regarding the presence of *P. levii* in bovine NHL, Staton et al. (36) identified *P. endodontalis* [which is highly associated with chronic oral infections in humans, (37)] by PCR in the vast majority of BDD-WLD and BDD-SU lesions. Thus, it was suggested that *P. endodontalis* could chiefly contribute to the development of NHL. Since *P. levii* was detected in three out of four NHL lesions as revealed by a strong hybridization signal, the assumption is supported that *P. levii* might indeed have a pathobiological role in BDD onset and progression. Further studies on higher numbers of NHL cases are needed to understand the role of *P. levii* in this and other types of claw diseases.

*F. necrophorum* was frequently found in BDD-WLD, BDD-SU, and BDD-associated toe necrosis in a previous study (36). These findings contrast with our results, as we did not detect *F. necrophorum* in the investigated NHL lesions. We hence question the proposed theory on a causative role of *F. necrophorum* in the pathogenesis of NHL.

*D. nodosus* was only detected in one case of BDD-HFA, and the hybridization signal was moderate. This finding is in line with the observations by Staton et al. (36) who detected these bacteria in only 2/10 NHL lesions screened. While sheep are the primary hosts for benign and virulent strains of *D. nodosus* involved in the multifactorial pathogenesis of ovine footrot (38), cattle typically host benign *D. nodosus* strains (39). Therefore, a relevant role in the development of NHL seems unlikely.

One main advantage of FISH as a culture-independent molecular biological method compared to PCR techniques is that it can localize bacterial infection. Using FISH, a distinction can be made between superficial colonization and invasion of deeper epidermal layers, and well-established infection scoring methods can be used (18, 25). Therefore, FISH constitutes a valuable tool to assess the involvement of different pathogens in the development of NHL, especially in combination with highly sensitive amplification techniques.

In conclusion, *P. levii* was detected in three of four cases of NHL, suggesting a potential synergistic activity of *P. levii* and BDD-associated treponemes in the development of bovine NHL. In contrast, present results do not allow a central role to be assigned to *F. necrophorum* or *D. nodosus* in the pathogenesis of NHL. The synergistic activity of *P. levii* and BDD-associated treponemes in the development of bovine NHL should be further investigated by the inclusion of a higher number of cases to show a consolidated association. Still, the results presented in this report provide new insights into the etiopathogenesis of NHL and encourage further research on this relevant claw disease.

## Data availability statement

The original contributions presented in the study are included in the article/Supplementary materials, further inquiries can be directed to the corresponding author.

## Ethics statement

Ethical review and approval was not required for the animal study because lesional tissue was obtained in the course of therapeutic debridement. Written informed consent was obtained from the owners for the participation of their animals in this study.

## Author contributions

MA was responsible for data collection, molecular analyses, and writing the first draft of the manuscript. ES and DD supported the data collection. CG performed the histological analyses. TJ and SB supported the data analyses. AS, SB, and JW edited the manuscript. AS supervised the study. All authors contributed to the manuscript and approved the final version.

## Funding

This work was supported by the Heard Health Management Initiator Grant, Institute of Animal Pathology and Clinic for Ruminants (Vetsuisse Faculty, University of Bern, Switzerland).

## Conflict of interest

The authors declare that the research was conducted in the absence of any commercial or financial relationships that could be construed as a potential conflict of interest.

## Publisher's note

All claims expressed in this article are solely those of the authors and do not necessarily represent those of their affiliated organizations, or those of the publisher, the editors and the reviewers. Any product that may be evaluated in this article, or claim that may be made by its manufacturer, is not guaranteed or endorsed by the publisher.
