# Supplementary material for: “Non-healing” claw horn lesions in dairy cows: Clinical, histopathological and molecular biological characterization of four cases
Source: Front Vet Sci. 2022 Oct 19;9:1041215. doi: 10.3389/fvets.2022.1041215 (PMC9627347; doi:10.3389/fvets.2022.1041215)
Supplement: Supplementary file 2 [file Table_2.DOC]

Supplementary Material

| **Phylotype /**  **Species** | **Oligonucleotide probes** | **Sequence** | **Reference** |
| --- | --- | --- | --- |
| Bacteria | S-D-eub-338 | 5´-GTC ATT CCA TCG AAA CAT A-3´ | (1) |
| *Treponema* spp. | S-S-TrepGenus-725 | 5´-CAG AAA CYC GCC TTC GCC-3´ | (2) |
| *T. medium* | S-S-Trep-I:B:C7-432 | 5´-CAT CAG ATG AGC ATT CCC-3´ | (2) |
| *T. phagedenis* | PT6 | 5'-CA TCA AGG ACG CAT TCC CTC-3' | (3) |
| *T. pedis* | T. pedis | 5'-AG AGT CCT CAA CCT TTA CGT GTT-3' | (3) |
| *T. refringens* | Trep. refringens | 5'-GC TCC CTT TCC TTA CAT GAT-3' | (3) |
| *D. nodosus* | S-S-D.nodosus-443 | 5'-CAT GCA CCG TTC TTC ACT-3' | (3) |
| *F. nechrophorum* | S-S-F.necrop-183 | 5'-GAT TCC TCC ATG CGA AAA-3' | (3) |
| *P. levii* | P. levii-443 | 5'-TACCTACGTTTACTCGCC-3' | (4) |

**Table S1.** Names and sequences of 16S rRNA-targeting oligonucleotide probes used in this study.

**Reference List**

1. Amann RI, Ludwig W, Schleifer KH. 1995. Phylogenetic identification and in situ detection of individual microbial cells without cultivation. Microbiol. Rev. 59, 143-169.
2. Klitgaard K, Boye M, Capion N, Jensen TK. 2008. Evidence of multiple *Treponema* phylotypes involved in bovine digital dermatitis as shown by 16S rDNA analysis and fluorescent *in situ* hybridisation. J. Clin. Microbiol. 46, 3012-3020.
3. Rasmussen M, Capion N, Klitgaard K, Rogdo T, Fjeldaas T, Boye M, Jensen TK. 2012. Bovine digital dermatitis: Possible pathogenic consortium consisting of *Dichelobacter nodosus* and multiple *Treponema* species. Vet. Microbiol. 160, 151-161.
4. Nielsen MW, Strube ML, Isbrand A, Al-Medrasi WDHM, Boye M, Jensen TK, et al. 2016. Potential bacterial core species associated with digital dermatitis in cattle herds identified by molecular profiling of interdigital skin samples. Vet. Microbiol. 186, 139–149.
